# The possibility of spatial mapping of soil organic carbon content at three depths using easy-to-obtain ancillary data in a Mediterranean area

**DOI:** 10.12688/openreseurope.14716.1

**Published:** 2022-09-20

**Authors:** Francisco José Blanco Velázquez, Mahmoud Shahabi, Hossein Rezaei, Félix González-Peñaloza, Farzin Shahbazi, María Anaya-Romero

**Affiliations:** 1 Evenor-Tech, Seville, Andalusia, 41018, Spain; 2 Soil Science Department, Faculty of Agriculture, University of Tabriz, Tabriz, Iran

**Keywords:** Digital soil mapping, Environmental covariates, organic carbon, uncertainty analysis

## Abstract

**Background:** Unlike most of Europe, Andalucía in southern Spain as a Mediterranean area still lacks digital maps of soil organic carbon (SOC) content at multiple depths, which can be generated by machine learning algorithms. The wide diversity of climate, geology, hydrology, landscape, topography, vegetation, and micro-relief data as easy-to-obtain covariates has facilitated the development of digital soil mapping (DSM). The purpose of this research is to model and map the spatial distribution of SOC at three depths, in an area of approximately 10000 km ^2^ located in Seville and Cordoba Provinces, and to use R programming to compare two machine learning techniques (cubist and random forest) for developing SOC maps at multiple depths.

**Methods:** Environmental covariates used in this research include nine derivatives from digital elevation models (DEM), three climatic variables, and 18 remotely-sensed spectral data (band ratios calculated by Landsat-8 Operational Land Imager ‘OLI’ and Sentinel-2A Multispectral Instrument ‘MSI’ in July 2019). In total, 300 soil samples from 100 points at three depths (0–25 cm, 25–50 cm, and 50–75 cm) were taken from existing literature. Both machine learning techniques were compared taking into account their accuracy using the goodness-of-fit criteria to predict SOC.

**Results:** The findings showed that integrating the indices derived by Landsat-8 OLI and Sentinel-2A MSI satellite data had a better result than when satellite data was used separately.

**Conclusions:** We obtained evidence that the resolution of satellite images is a key parameter in modelling and digital mapping.

## Introduction

The knowledge of the spatial and vertical distribution of soil organic carbon (SOC) is helpful either in optimizing management for soil fertility or in monitoring the potential of carbon sequestration in subsoil horizons ( Lorenz & Lal, 2005). The EU Commission is working on the future EU Soil Strategy whose objectives include protecting soil fertility, reducing soil erosion, increasing soil organic matter and restoring carbon-rich ecosystems ( EC, 2021). SOC is one of the soil properties which will determine the vegetation suitable for growing on that soil. In addition, this vegetation will influence the amount and vertical distribution and forms of SOC in the soil ( Bailey *et al*., 2019).

There is a large variation of SOC at field scale due to its dependency on environmental variables. Moreover, it is necessary to develop digital maps in an accurate manner using cost-free indices over the area of interest. A compilation of relevant covariates ( *scorpan* factors) can be found in Minasny *et al*., 2013, related to SOC. McBratney *et al*. (2003) reported that digital elevation model (DEM) and spectral reflectance bands from satellite imageries were commonly used to handle digital soil mapping (DSM). An accurate prediction of SOC using solely easy-to-obtain indices e.g., DEM-derived data can save considerable labour and costs ( She *et al*., 2014). The spatial patterns of SOC were linked topography in a case study conducted in south-eastern Spain ( Schwanghart & Jarmer, 2011). In addition, SOC can be successfully predicted by using remotely-sensed data ( Odebiri *et al*., 2020). It was also reported by Lamichhane *et al*. (2019) that climate is an influential factor in predictive mapping of SOC level at regional scales, followed by parent materials, topography and land use. Venter *et al*. (2021) relates elevation, mean annual precipitation, leaf area index (LAI) standard deviation, temperature wettest quarter and topographic diversity index as the variables of most importance for predicting SOC through Random Forest (RF) in South Africa. Furthermore, there are other research works with Landsat-8 OLI, Sentinel-2A and EnMap spectral bands to estimate soil organic matter ( Rosero-Vlasova *et al*, 2019) in Brazil.

In summary, DSM has shifted from a research phase into practical usage ( Minasny & McBratney, 2016). One of the most important pathways relevant to the applied DSM is digital soil assessment (DSA) which was suggested by Carré *et al*. (2007). In DSA, the understanding of the spatial prediction of soil attributes can relate to many other parameters e.g., potential distribution of oaks in southern Spain ( Pino-Mejías *et al*., 2010); agriculturally important nutrients ( Shahbazi *et al*., 2019a) and the soil ripening process in Iran ( Mousavi *et al*., 2020); and available water content in Australia ( Gooley *et al*., 2014).

Contrary to conventional soil mapping, DSM uses quantitative inference models to generate predictions of soil properties in a geographic database ( Zeraatpisheh *et al*., 2020). To avoid a lot of soil surveying and sampling for mapping the spatial distribution of each property, it can be successfully conducted using easy-to-obtain indices as an envoy of aforementioned factors ( Shahbazi *et al*., 2019b). Data mining or machine learning techniques are popular for mapping soil properties with the application of numerous spatially explicit covariates ( Blanco-Velázquez *et al*., 2019). In this way, the literacy of global positioning systems (GPS), geographic information systems (GIS), acquisition of remotely-sensed data, terrain-related attributes, inference models, and software for data analysis e.g. R programming language are essential ( Malone *et al*., 2017).

In terms of SOC, the literature revealed that various environmental covariates as well as different data mining techniques have been applied worldwide ( Muñoz-Rojas *et al*., 2013 and Muñoz-Rojas *et al*., 2012). Taghizadeh-Mehrjardi *et al*. (2016) reported that artificial neural networks (ANN) have the highest performance in prediction of SOC in Iran using ancillary data variables derived from a DEM and Landsat 8 images. A review by Lamichhane *et al*. (2019) showed that RF was the better-fitting model than multiple linear regression and other machine learning techniques in most comparative studies. While, Rentschler *et al*. (2020) highlighted that the spatial prediction of SOC with cubist provided slightly lower error than with RF in the north of Leipzig, Saxony, Germany. It was generally recognised that application of various techniques will result in different importance of predictors in DSM of continuous attributes for a specific study area ( Piikki *et al*., 2021).

Based on the literature, this research evaluates two machine learning techniques, namely cubist and RF, as candidates for prediction of SOC in southern Spain (Seville) using a set of environmental covariates derived by Landsat-8 OLI, Sentinel-2A Multispectral Instrumental (MSI), DEM, and climate factors such as temperature and precipitation. CONSOLE (Contract Solutions for Effective and lasting delivery of agri-environmental-climate public goods by EU agriculture and forestry, GA 817949) is a H2020 project which focuses on promoting the delivery of Agri-Environmental Climate Public Goods by agriculture and forestry through the development of improving contractual solutions. In the framework of this project, we have selected olive groves under integrated production as a contract solution that promotes climate regulation through increase of soil organic carbon sequestration.

The aims of this study are: 1) Identify and map SOC at three depths (0–25 cm, 25–50 cm, and 50–75 cm) using satellite multispectral images (Landsat-8 OLI, Sentinel-2A MSI) as well as integration and terrain-related attributes (DEM-derived data), and climatic variables (temperature, precipitation and evapotranspiration) in a Mediterranean area (Seville, southern Spain); 2) Compare the capability of the cubist and RF models in mapping the vertical and spatial distribution of SOC and; 3)Describe the ranking of variables importance in prediction of SOC at multiple depths using the parsimonious model across the study area.

## Methods

### Area description and summary of methods

**Figure 1.**
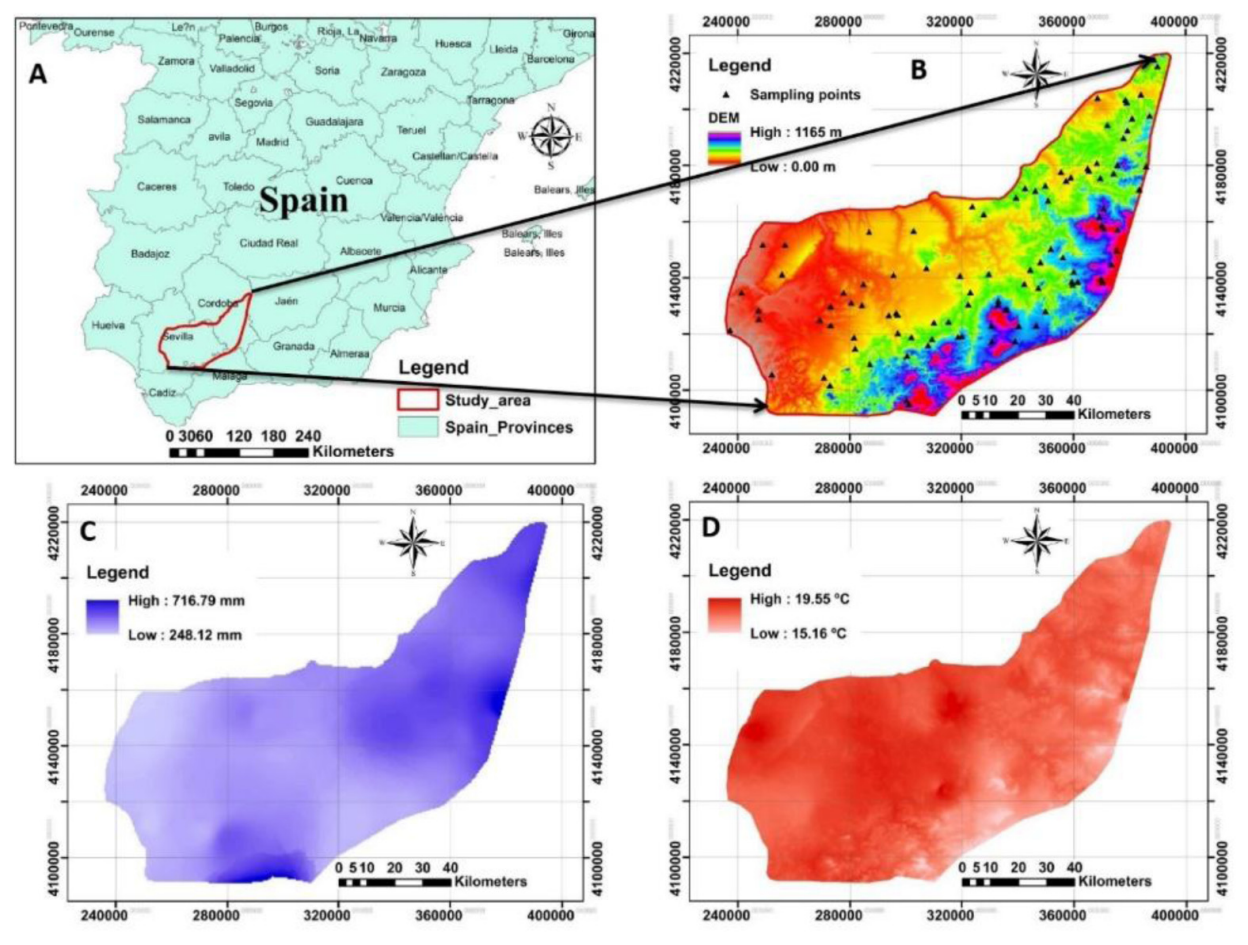
The location and outline of the study area. **A**: Represents the study area in Spain; **B**: the digital elevation model (DEM) of the study area associated with sampling points; **C** and **D**: represent the spatial maps of precipitation and temperature respectively measured for the 10 last consecutive years across the study area. (Own source).

The study area is located in Seville, southern Spain, which has a Mediterranean climate. It has a dry summer and mild, wet winter and covers an area of 10000 km ^2^. The altitude varies from 0 to 1165 m above sea level ( Figure 1). Also, the mean annual precipitation, temperature and evapotranspiration for the last 10 consecutive years were measured by the regional administration as 248 mm to 716 mm, 15 °C to 20 °C and 624 mm to 829 mm, respectively ( REDIAM, 2021). The olive groves under integrated production are one of the main land use types in the entire study area. Integrated production is an agricultural system of production of plants using farming techniques that ensures sustainable agriculture, using methods of integrated pest management compatible with environmental protection, agricultural productivity and the use of natural production mechanisms and resources described in Hinojosa-Rodríguez *et al.* (2014).

**Figure 2.**
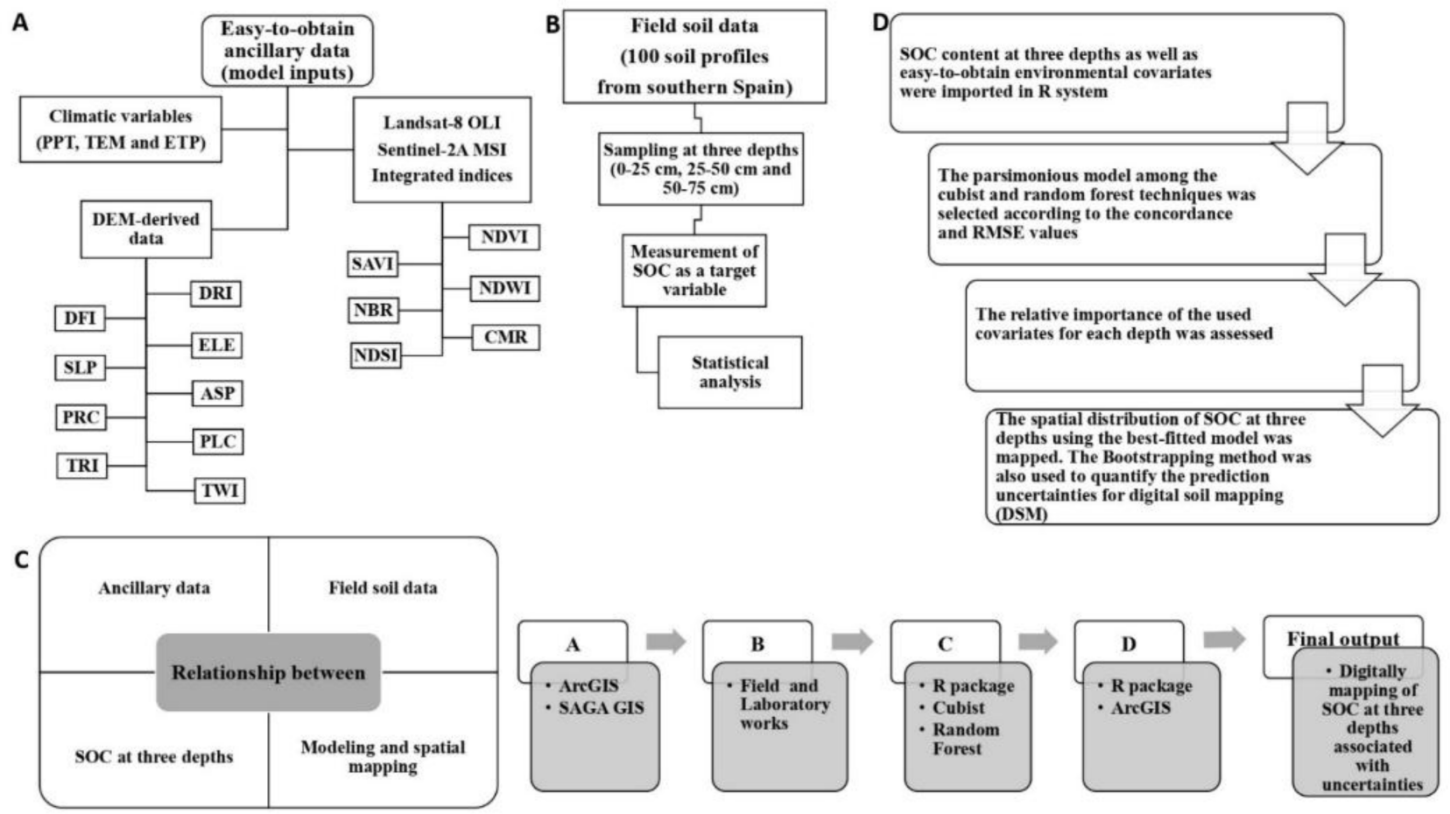
Simplified flowchart of the research in this study. SOC: Soil organic carbon (%); PPT: precipitation; TEM: temperature; ETP: Evapotranspiration; DRI: direct insolation; DFI: diffuse insolation; ELE: elevation; SLP: slope; ASP: aspect; PRC: profile curvature; PLC: plan curvature; TRI: terrain ruggedness index; TWI: topographic wetness index; NDVI: normalized difference vegetation index; SAVI: soil adjusted total vegetation index; NDWI: normalized difference water index; NBR: normalized burn ratio; CMR: clay minerals ratio; NDSI: normalized difference salinity index.

Integrated production includes several rules ( Romero-Gámez *et al.*, 2017) for crop, soil and water management in order to increase the sustainability production and other agri-environmental public goods, such as carbon sequestration, and promoting the reduction of fertilizers and pesticides. To facilitate the employed procedures across this research, the flowchart is represented in Figure 2.

### Field sampling and soil analysis

The first step was compiling all data available for Seville, southern Spain from 1990–2019. For that, three data sources were analysed: 1) REDIAM (Regional Environmental database; 2) SEISNET: Spanish Soil Information System on the Internet and; 3) ASAJA-Sevilla: farmer association from Seville that collect data from farmers and who provided it directly to the authors.

The information compiled (physical and chemical soil variables (SEISNET and ASAJA-Sevilla); site, mean annual precipitation, temperature and evapotranspiration (REDIAM)) were harmonised using R Software (identifying potential duplicates and errors) so that they had the same resolution and extent for analysing the relationship between SOC and other variables in-situ and ex-situ. A total of 22 commercial olive groves included in the data compiled from ASAJA-Sevilla were selected, taking into account their representativeness in terms of soil types and climatic conditions.

SOC at three depths (0–25 cm, 25–50 cm, and 50–75 cm) was measured in the laboratory in previous research/technical works (this information can be found in the metadata in SEISNET (Information level #3) by wet oxidation with chromic acid and back titration with ferrous ammonium sulphate according to the methods outlined in Nelson and Sommers (1996).

### Statistical analysis

Since RF and cubist were used to model and map the spatial distribution of SOC, and the data do not need to be normally distributed ( Svetnik *et al*., 2003), the original data were employed in modelling. However, seven statistical criteria e.g., min, max, mean, standard deviation, coefficient of variation, skewness and kurtosis were calculated for the data set. The aforementioned basic statistics were performed using the package of *“fBasics”* with R language ( Wuertz *et al.*, 2017).

### Ancillary data

Due to variation on elevation, land uses, parent material and even climate, the spatial distribution of SOC at three depths is likely to be predicted as some function of given ancillary data. For this purpose, to gain all required factors identified by *scorpan* model, a DEM, Landsat-8 OLI data and Sentinel-2A MSI imageries spectral data were obtained via USGS-EROS (see *Underlying data*). Climatic variables (precipitation, temperature and evapotranspiration) were also used in this study (obtained from REDIAM, see *Underlying data*) ( Table 1). Since all covariates (a total number of 30) have a different resolution, those were initially aligned to the same grid cell resolution and extent as exemplified by Malone *et al*. (2017). Here, a 30m grid was used. The coordinate reference system used in this study was WGS1984 UTM Zone 30.

**Table 1.** The specifications of used indices derived by DEM and remote sensing imageries.

Index	Abbreviation	Description	Derived by	Details and formulation
Direct insolation	DRI	Topography-Potential incoming solar radiation	DEM	Terrain analysis, lightening, visibility
Diffuse insolation	DFI			
Elevation	ELE	Topography-Morphometry	DEM	Terrain analysis
Slope	SLP			
Aspect	ASP			
Profile curvature	PRC			
Plan curvature	PLC			
Terrain ruggedness index	TRI			
Topographic wetness index	TWI	Topography-Hydrology	DEM	Terrain analysis
Normalized difference vegetation index	NDVI_LST	Vegetation	Landsat-8 OLI	$NDVI=\frac{\left(NIR-Red\right)}{\left(NIR+Red\right)}$
	NDVI_SEN		Sentinel-2 MSI	
	NDVI_ITG		Integrated index	$(\frac{NDVI_{LST}+NDVI_{SEN}}{2}$
Soil Adjusted Total Vegetation Index	SAVI_LST	Vegetation	Landsat-8 OLI	$SAVI=\left(\frac{\left(NIR-Red\right)}{NIR+Red+L^{a})}\right)\times (1+L)$
	SAVI_SEN		Sentinel-2 MSI	
	SAVI_ITG		Integrated index	$(\frac{SAVI_{LST}+SAVI_{SEN}}{2}$
Normalized Difference Water Index	SAVI_LST	Vegetation	Landsat-8 OLI	$NDWI=\frac{\left(Green-NIR\right)}{\left(Green+NIR\right)}$
	SAVI_SEN		Sentinel-2 MSI	
	SAVI_ITG		Integrated index	$(\frac{NDWI_{LST}+NDWI_{SEN}}{2}$
Normalized Burn Ratio	NBR_LST	Landscape	Landsat-8 OLI	$NBR=\frac{\left(NIR-SWIR2\right)}{\left(NIR+SWIR2\right)}$
	NBR_SEN		Sentinel-2 MSI	
	NBR_ITG		Integrated index	$(\frac{NBR_{LST}+NBR_{SEN}}{2}$
Clay Minerals Ratio	CMR_LST	Geology	Landsat-8 OLI	$CMR=\frac{SWIR1}{SWIR2}$
	CMR_SEN		Sentinel-2 MSI	
	CMR_ITG		Integrated index	$(\frac{CMR_{LST}+CMR_{SEN}}{2}$
Normalized Difference Salinity Index	NDSI_LST	Geology	Landsat-8 OLI	$NDSI=\frac{\left(Red-NIR\right)}{\left(Red+NIR\right)}$
	NDSI_SEN		Sentinel-2 MSI	
	NDSI_ITG		Integrated index	$(\frac{NDSI_{LST}+NDSI_{SEN}}{2}$
Precipitation	PPT	Climate	information (climate) layers of Andalusia, Spain https://bit.ly/3CX7zOM https://bit.ly/3zS30Dm	
Temperature	TEM			
Evapotranspiration	ETP			

^a^ The L value varies depending on the amount of green vegetative cover (L=0.5)

***DEM-derived data*.** The terrain-related attributes were derived from the original DEM layer at a resolution of 30 m using various functions (slope gradients, profile, planform curvatures and so on) made available in SAGA GIS version 3.5.1 ( Conrad *et al*., 2015). The terrain analysis allows accurate routing of surface and subsurface flow through the landscape ( Ranatunga *et al*., 2008) which can be rapidly made with application of SAGA GIS. In this research, in addition to elevation (ELE) which was directly derived by DEM, eight derivatives were also classified under three broad categories. 1) Lighting or visibility: with derivatives including potential incoming solar radiation (direct and diffuse insolation abbreviated by DIR and DFI, respectively). 2) Morphometry: with derivatives including slope (SLP), aspect (ASP), curvature (plan and profile abbreviated by PLC and PRC, respectively), and terrain ruggedness index (TRI). 3) Hydrology: includes the topographic wetness index (TWI).

The DEM-derivatives relevant to morphometry e.g., SLP, ASP, PLC, PRC and TRI were numerously used in digital mapping of SOC ( She *et al*., 2014). Kravchenko & Bullock (2000) have defined these indices in a detailed manner.

The third category identifying the terrain-related attributes is hydrology. Topographic wetness index (TWI) was used in this work. Schwanghart and Jarmer (2011) found that there was a positive correlation between TWI and SOC even on steep slopes, and that correlation is not significant on wide pediments. To manually gain the TWI, it is necessary to calculate the specific upslope contributing area and slope while it was easily earned using SAGA GIS across the study area.

***Remotely-sensed data*.** Landsat-8 OLI and Sentinel-2A MSI images acquired in July 2019 (see *Underlying data*) were selected for further analysis in this project due to the high probability of no clouds in the images. Fortunately, the selected scenes comprise minimal cloud coverage and maximum soil surface exposure. On contrary of the previously performed research (such as Shahbazi *et al*., 2019a), the individual bands and Principal Component Analyses (PCA) of bands were not used here. To explain the vegetation, soil and water, landscape as well as geology of the study area, six band ratios were calculated using both images as well as their integrations for each index. The hypothesis of integrating the indices has been raised from the report of Wang *et al*. (2020) which demonstrated that combining Landsat TM and ALOS PALSAR images could predict more accurate SOC content, especially for soils with high vegetation canopy density in Spain. Those were normalized difference vegetation index (NDVI) ( Rouse *et al*., 1973), soil adjusted vegetation index (SAVI) ( Huete, 1988), normalized difference water index (NDWI) ( McFeeters, 1996), normalized burn ratio (NBR) ( Lanorte *et al*., 2013), clay minerals ratio (CMR) ( Drury, 2016), and normalized difference salinity index (NDSI) ( Allbed & Kumar, 2013). 

***Climatic variables*.** To predict the spatial distribution of SOC, it was found that despite topographic variables which had higher influence at finer scales, the climatic variables were more important at coarser scales e.g., sub-regional to global scales ( Adhikari *et al*., 2020). Among the previously described indices, climate variables contributed the most to explaining SOC content variation ( Zhou *et al*., 2020). Literature revealed that climate significantly controls the spatial pattern of SOC and its stock in soil. Therefore, precipitation, temperature and evapotranspiration at a 100-m resolution were used as the main attributes in prediction of SOC across the study area.

### Machine learning techniques

Regarding the accelerated adoption of machine learning (ML) techniques in prediction of soil properties, especially in the last 10 years ( Padarian *et al*., 2020), two methods, namely cubist and random forest (RF) were tested within R programming (version 3.5.1) for the prediction of SOC at three depths entire study area using our field soil data set and environmental covariates. Compared with the cubist, the difference between lower and upper quartiles in the RF is larger. Furthermore, RF tends to perform better in different iterations ( Zhou *et al*., 2019). Khaledian and Miller (2020) reported that the results taken by cubist are more interpretable than RFs’ which are semi-interpretable. We therefore performed both models, then one of them was selected as a parsimonious model to be applied in spatial mapping. The bootstrap aggregation or bagging that reduces variance within a noisy data set ( Breiman, 1996), was also set on 200 in this work. In this way the stability and accuracy of both models will be improved. The efficiency of tested models was evaluated in a calibration and validation data set (composed by 20% of data no included in the modelling process) using the goodness-of-fit criteria e.g., concordance (CCC) and root mean square error (RMSE). It is clear that R ^2^ measures the precision of the relationship (between observed and predicted) while concordance as a single statistic evaluates the accuracy and precision of the relationship. Similar work was fully explained by Malone *et al*. (2017) using the “ *goof*” function within the “ *ithir*” package in R ( Malone, 2016). 

***Cubist*.** Cubist is a rule-based model that has been numerously applied for prediction of SOC ( Akpa *et al*., 2016; Miller *et al*., 2015) with success. The tuning parameters in the cubist model are committees and neighbours. Regarding these parameters, it may improve the prediction performance due to its ability to “mine” non-linear relationships in data. The path through the tree was collapsed to create rules using boosting training. The rules, extrapolation and committee were set on 5, 10, and 5, respectively in the used script with the “ *Cubist*” package ( Kuhn & Quinlan, 2018). 

***Random forest*.** The other most commonly used ML technique in prediction of SOC is RF ( Dharumarajan *et al*., 2017; Grimm *et al*., 2008; Wiesmeier *et al*., 2011). Mapping using RF creates the spatial distribution of SOC at three depths in a quicker way because fitting a RF model in R is relatively straightforward. The tuning parameter in the RF model is the number of trees ( Liaw & Wiener, 2002) which were set on 1000 in this research using the package of “ *randomForest*” in R programming. In this technique, a group of observations from the training dataset (20% of data) was selected and built a decision tree associated with those selected observations. The calibration and validation of the model were derived using in-the-bag and out-of-bag (OOB). A total of 200 bootstrap samples were supplied to model due to setting the bags on 200 iterations. Since the arrangement of 30 used environmental covariates is randomly ordered, all possibilities were therefore considered in selection of covariates.

### Acquiring spatial maps

In addition to the rich capacity of modelling in R, it has a user-friendly environment to map spatial data ( Malone *et al*., 2017). Bivand *et al*. (2013) documented some methods for handling spatial data and analysis in R in a detailed manner. The spatial mapping was facilitated using the “ *ggplot2*” package in R ( Wickham, 2016) as a way to create codes that make sense to the user. It is obvious that both point data and covariates should have the same projection in advance to be easily import, view, and export points and rasters to, in, and from a GIS. The final digital maps ( Figure 6– Figure 8) will be as the main output for further visualization as well as interpretation in digital assessments. 

### Uncertainty analysis

Arrouays *et al*. (2020) reported that uncertainty maps may give very strong arguments in DSM because the identifying missing soil data and excessive field work will be distinguished. On the other hand, the quantified uncertainty analysis is essential to be associated with digital maps because they come with some errors ( Wadoux *et al*., 2020). Various methods depending on the objectives were used in such analysis e.g., Monte Carlo ( Tan *et al*., 2014); Bayesian Network ( Stritih *et al*., 2019); and computing a vector total of RMSE ( Weng, 2002).

In our research, the next step was to calculate mean values by taking the average of all the simulated predictions at each pixel using the base function “ *mean*” to a given stack of rasters. Since there is no simple function to estimate the variance, the sum of square differences was firstly calculated at each pixel from the prediction maps. The maps of prediction interval (PI) range for SOC at three depths were also provided by calculating the difference between upper and lower 95% limit of predictions ( Xiong *et al*., 2015). The high value of PI at any pixel demonstrates the low level of confidence. For further visualisation, the standard deviation and standard error of predictions at any depth were also associated with each digital map.

## Results and discussion

### Brief soil data

**Figure 3.**
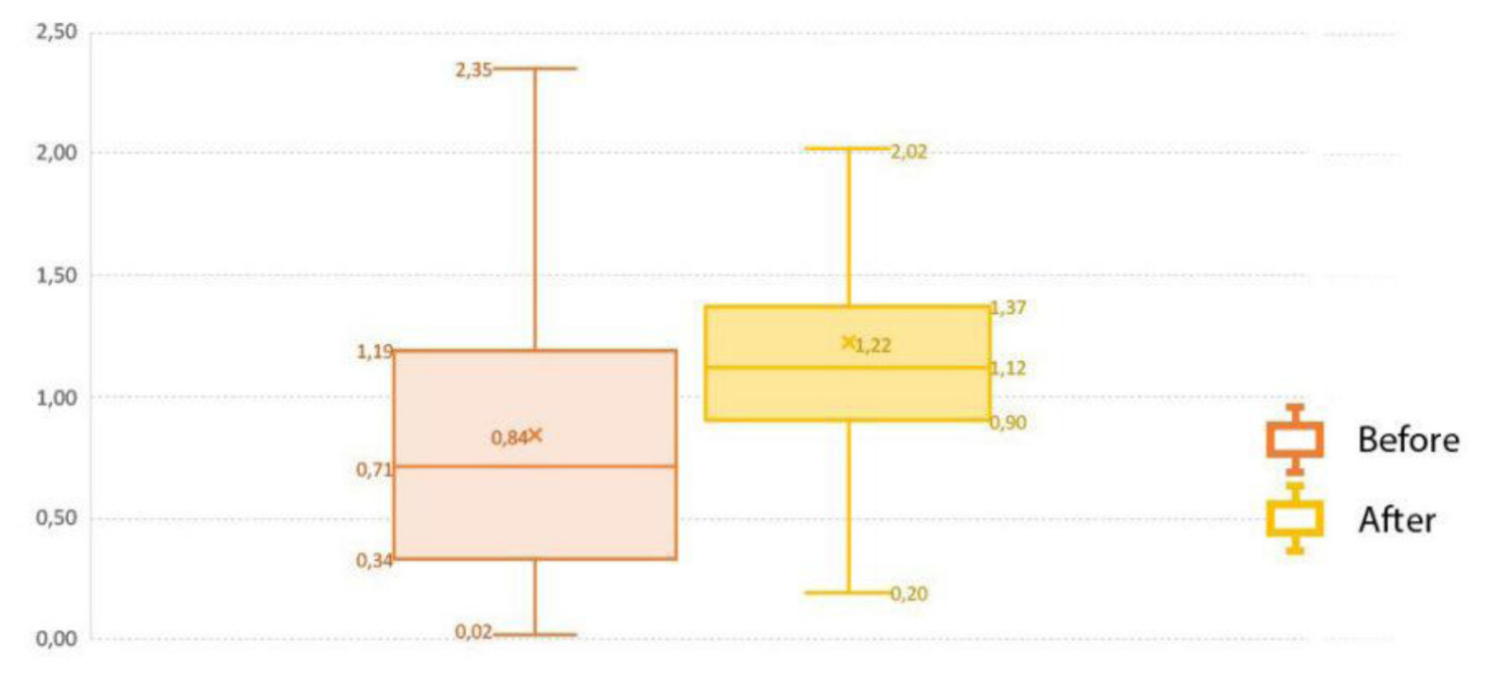
Comparison of soil organic carbon before (1990–2000) from REDIAM and after (2010–2020) from ASAJA-Sevilla applying the Integrated Production to representative Mediterranean benchmark soils under olive crop.

The soil data compiled across the study area illustrates OC differences among soil depth ( Table 2). The results revealed that soils have a regular decrease in OC with depth which may be related to natural processes such as alluvial deposition. Caro Gomez *et al*. (2011) reported that alluvial episodes with associated lithic techno-complexes were identified across the Guadalquivir River valley (Seville, Spain). Furthermore, the highest CV (%) was recorded for SOC _50–75cm_, followed by SOC _25–50cm_ and SOC _0–25cm_. These variations may have occurred due to different soil types e.g., Vertisols, Nitisols, Luvisols, etc in the entire study area. In fact, the impact of Integrated Production was analysed comparing SOC data previous Integrated Production implementation (2005) and current SOC data. The results showed the increase in SOC due to the implementation of Integrated Production in terms of crop and soil management. ( Figure 3).

**Table 2.** The statistical descriptive of used field observations across the study area (n=100).

	min	max	mean	SD	CV (%)	skewness	kurtosis
SOC _0–25cm_	0.02	2.63	0.81	0.53	65.08	-0.29	0.99
SOC _25–50cm_	0.02	1.75	0.52	0.39	75.17	0.19	1.14
SOC _50–75cm_	0.01	1.63	0.26	0.38	147.51	1.00	1.50

SOC: soil organic carbon (%); SD: standard deviation; CV: coefficient of variation.

### Terrain-related attributes

**Figure 4.**
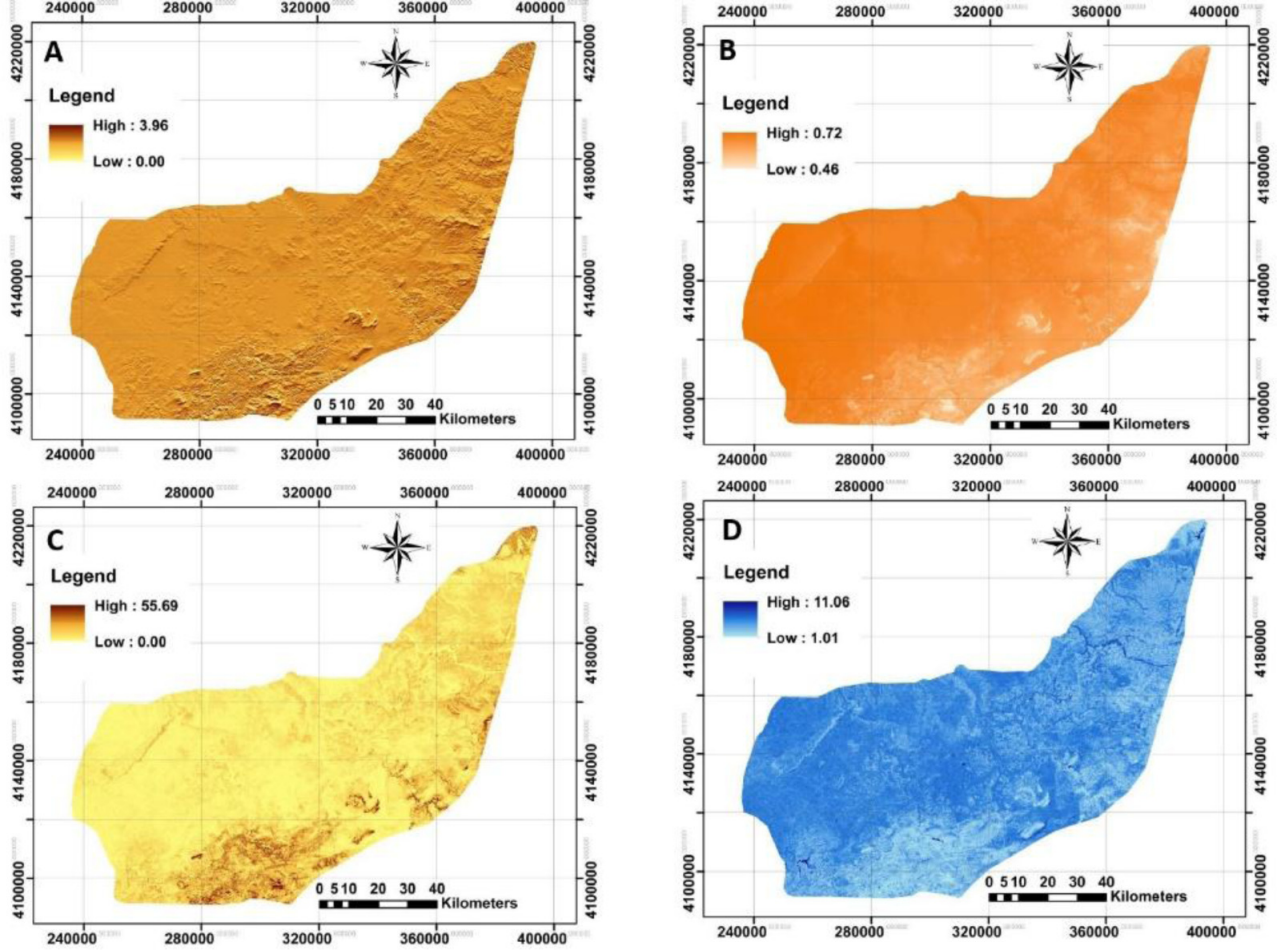
Four examples of applied terrain-related attributes to model across the study area. **A**: Direct insolation (DRI); **B**: Diffuse insolation (DFI); **C**: terrain ruggedness index (TRI); **D**: topographic wetness index (TWI). (Own source).

A total of nine DEM-derived data were supplied as predictors relevant to the lighting, morphometry and hydrology. Four examples of spatial distribution of DRI, DFI, TRI and TWI provided by SAGA GIS were presented in Figure 4 to facilitate in interpreting the study’s area condition. These covariates allow the analysis of SOC across case study areas and their relation with terrain attributes. In general, there was a different gradient of aforementioned covariates. DRI and DFI mean values varied from zero to 3.96 (1.66, on average) and 0.46 to 0.72 (0.70, on average). Also, the values of SD for both covariates were 0.29 and 0.01, respectively. In terms of TRI, it has been developed by Riley *et al*. (1999) as an index to quantify the topographic heterogeneity from level to extremely rugged by seven classes. It is interesting that TRI varied from zero to 55.69 indicating the level terrain surface across the study area. The mean value and SD were 1.82 and 1.55, respectively. The fourth studied covariate was TWI. It varies from 1.01 to 11.06 with a mean value of 5.43 across the study area. The ability of TWI in prediction of soil moisture and plant assemblages was recently reported by Kopecký *et al*. (2021). Similar observations were performed for the rest of the terrain-related attributes. 

### Remotely-sensed data

**Figure 5.**
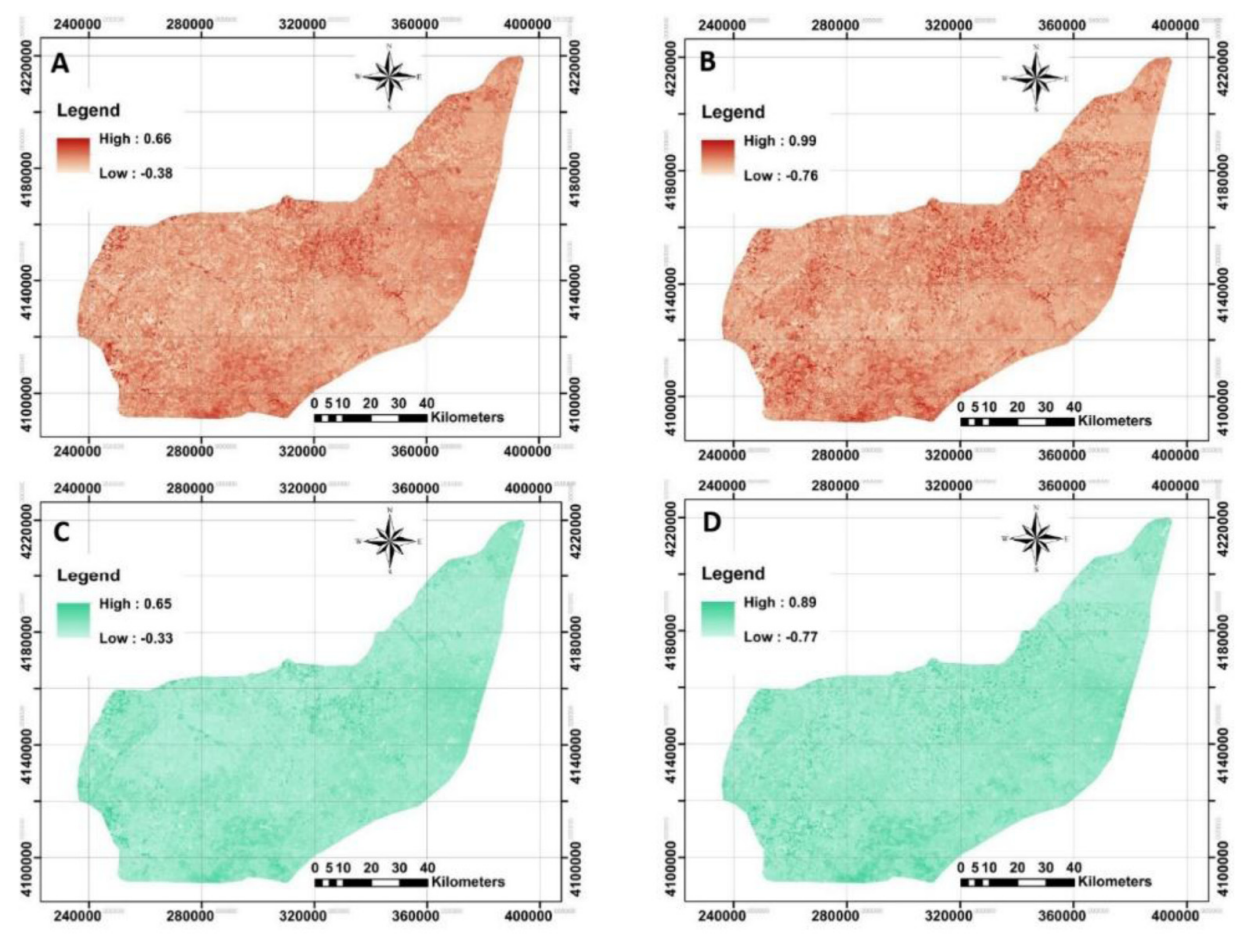
Four examples of applied Remote Sensing data using Landsat-8 OLI and Sentinel-2A MSI data to model across the study area. **A** and **B**: represent Normalized burn ratio (NBR) derived from Landsat-8 and Sentinel-2A, respectively; **C** and **D**: represent normalized difference vegetation index (NDVI) derived from Landsat-8 and Sentinel-2A, respectively. (Own source).

The next step was to acquire both Landsat-8 OLI and Sentinel-2A MSI imageries. To monitor the spatial distribution of each index calculated by each image, NBR and NDVI were illustrated in Figure 5.

The original raster files in QGIS showed that the calculated mean values for NBR derived from Landsat-8 and Sentinel-2A were no longer different (approximately 0.12, on average). However, Landsat-based NDVI did differ significantly from Sentinel-based NDVI. Further analysis by converting the NDVI maps from stretch to classified mode has provided an opportunity to identify bare soils, water and very low to high dense plant covers entire area. This idea was previously reported by Julien *et al*. (2011) in observation of land surface temperature for detecting changes in the Iberian land covers, Spain. The map of NDVI_LST strongly implies that about 0.2% of the study area has been categorized as the bare soil or water. The highest area (70%) was classified as very low cover (0<NDVI<0.2), followed by low cover (29%, 0.2<NDVI<0.4), moderately cover (0.7%, 0.4<NDVI<0.6) and moderately high cover (0.1%, 0.6<NDVI<0.8). Similar classification on the NDVI_SEN showed that 46%, 44%, 8% and 1% of the study area have very low, low, moderately low and moderately high cover, respectively. The results showed a significant increase in overall classification when the NDVI derived from Sentinel-2A compared to Landsat-8. One interpretation of this finding is that the launch of Sentinel-2A satellites has boosted the development of many applications that could benefit from the fine resolution of the supplied information ( Chakhar *et al*., 2020). These kinds of observations were performed for the remaining covariates, but are not fully presented here.

### Final machine learning technique

Machine learning allowed us to analysis the huge dataset and their relevance to SOC at multiple depths. For that, the use of open software like as R facilitated modeling and then mapping. For this study, the statistical criteria e.g., CCC, RMSE and bias for calibration and validation dataset using two machine leaning techniques were summarized in Table 3.

**Table 3.** The performance of candidate models for estimating SOC across the study area (n=100).

Calibration						
	Concordance		Root mean square error		Bias	
	Random Forest	Cubist	Random Forest	Cubist	Random Forest	Cubist
SOC _0-25cm_	0.86	0.32	0.22	0.46	0.00	-0.06
SOC _25-50cm_	0.88	0.53	0.15	0.29	0.00	-0.04
SOC _50-75cm_	0.85	0.43	0.16	0.31	0.00	-0.10
Validation							
SOC _0-25cm_	0.15	0.07	0.54	0.55	0.02	-0.08
SOC _25-50cm_	0.28	0.26	0.36	0.38	0.01	-0.04
SOC _50-75cm_	0.13	0.06	0.37	0.41	0.02	-0.09

SOC: soil organic carbon.

**Figure 6.**
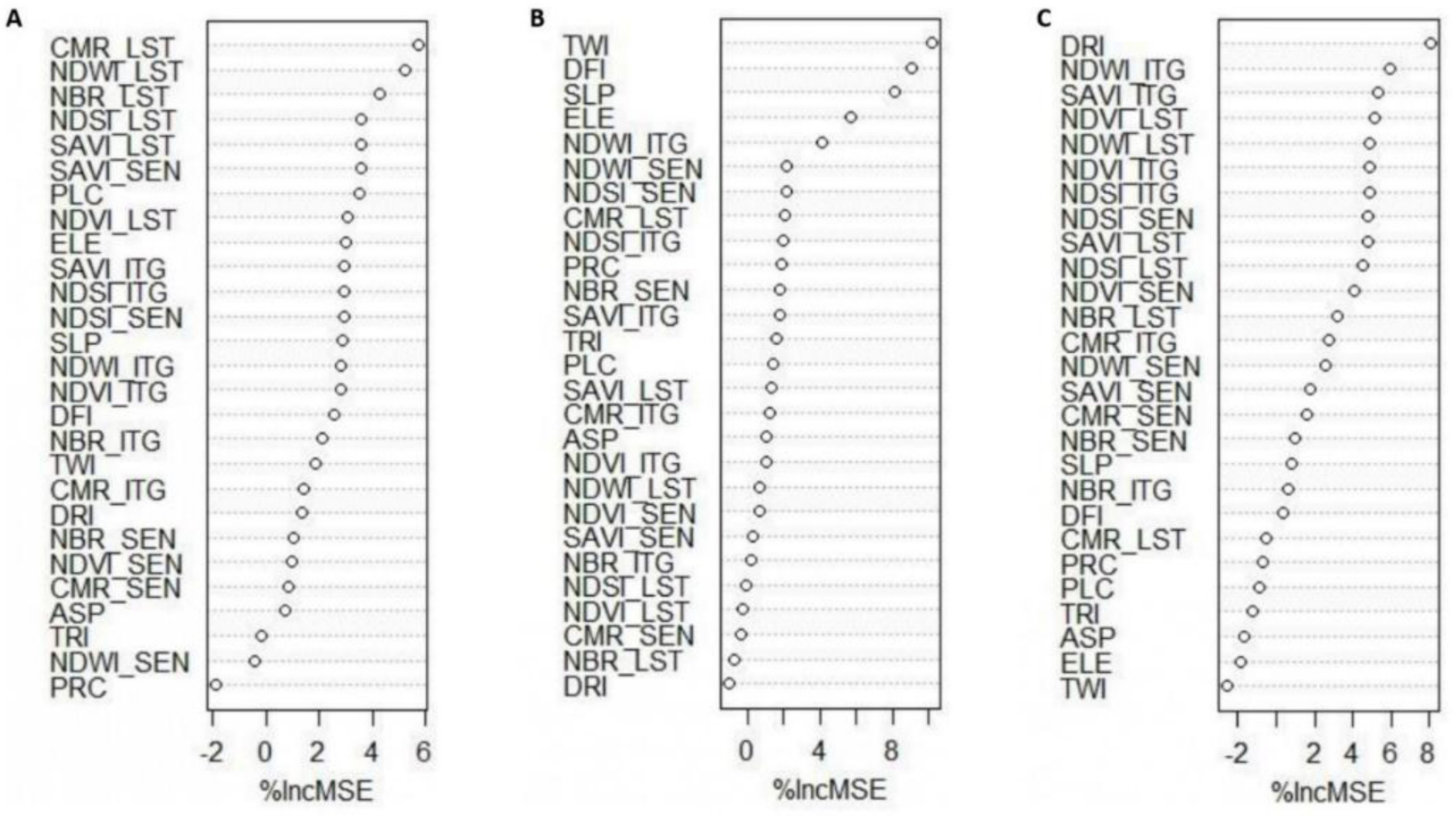
The importance ranking of used environmental covariates (n=30) for predicting SOC across the study area for any depth ( **A**: SOC0–25cm; **B**: SOC25–50cm; **C**: SOC50–75cm). _LST: Indices derived from Landsat-8 OLI; _SEN: indices derived from Sentinel-2A MSI; _ITG: An index illustrating the average of Landsat-8 OLI and Sentinel-2A MSI relevant to each index; PPT: precipitation; TEM: temperature; ETP: Evapotranspiration; DRI: direct insolation; DFI: diffuse insolation; ELE: elevation; SLP: slope; ASP: aspect; PRC: profile curvature; PLC: plan curvature; TRI: terrain ruggedness index; TWI: topographic wetness index; NDVI: normalized difference vegetation index; SAVI: soil adjusted total vegetation index; NDWI: normalized difference water index; NBR: normalized burn ratio; CMR: clay minerals ratio; NDSI: normalized difference salinity index.

Finally, we deduced that RF outperformed cubist in prediction accuracy. Since RF was selected as a good model in predicting, therefore, the importance of covariates was ranked for each layer ( Figure 6). The results generally revealed that Landsat -based indices are not better predictors at all studied depths. In addition, the climate data were not as important as other supplied covariates. A possible reason is that the topography and plant cover were more variable than climate over the study area. The integrated indices e.g., NBR_ITG, NDWI_ITG and CMR_ITG were identified as the most important covariates in prediction of SOC _0–25cm_, SOC _25–50cm_ and SOC _50–75cm_, respectively. These findings showed that the idea of combining indicators was an interesting idea and presented a good result. Similar work has been successfully conducted by Quintano *et al*. (2018) in Sierra de Gata (central-western Spain) for initial assessment of burn severity. The novelty of the approach was also reported by Singh *et al*. (2020) in water-use mapping. In terms of SOC _50–75cm_, it was found that integrated and Landsat-based indices in addition to DRI were important covariates. As well as, SAVI_SEN, NDWI_SEN and CMR_SEN showed good results in the three depths analysed. This remark may suggest that Sentinel data is the most influential data in the integrated indexes. In our view, the most compelling explanation for the present set of findings is that integrated-based indices were in the top ranks for SOC _0–25cm_ and SOC _50–75cm_. This finding may be explained by the idea that SOC content near the surface is linked with water availability or other dynamic properties, whereas the distribution of SOC with depth is linked with land use, site factors and temperature ( Hobley & Wilson, 2016).

**Figure 7.**
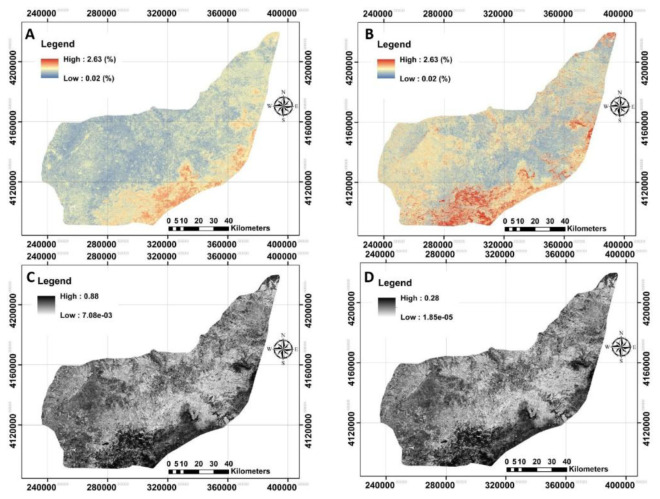
The provided digital maps of SOC0–25cm across the study area using RF model. **A**: Mean prediction (%); **B**: prediction of interval range (%); **C**: standard error map of the prediction; **D**: variance map of the prediction. (Own source).

**Figure 8.**
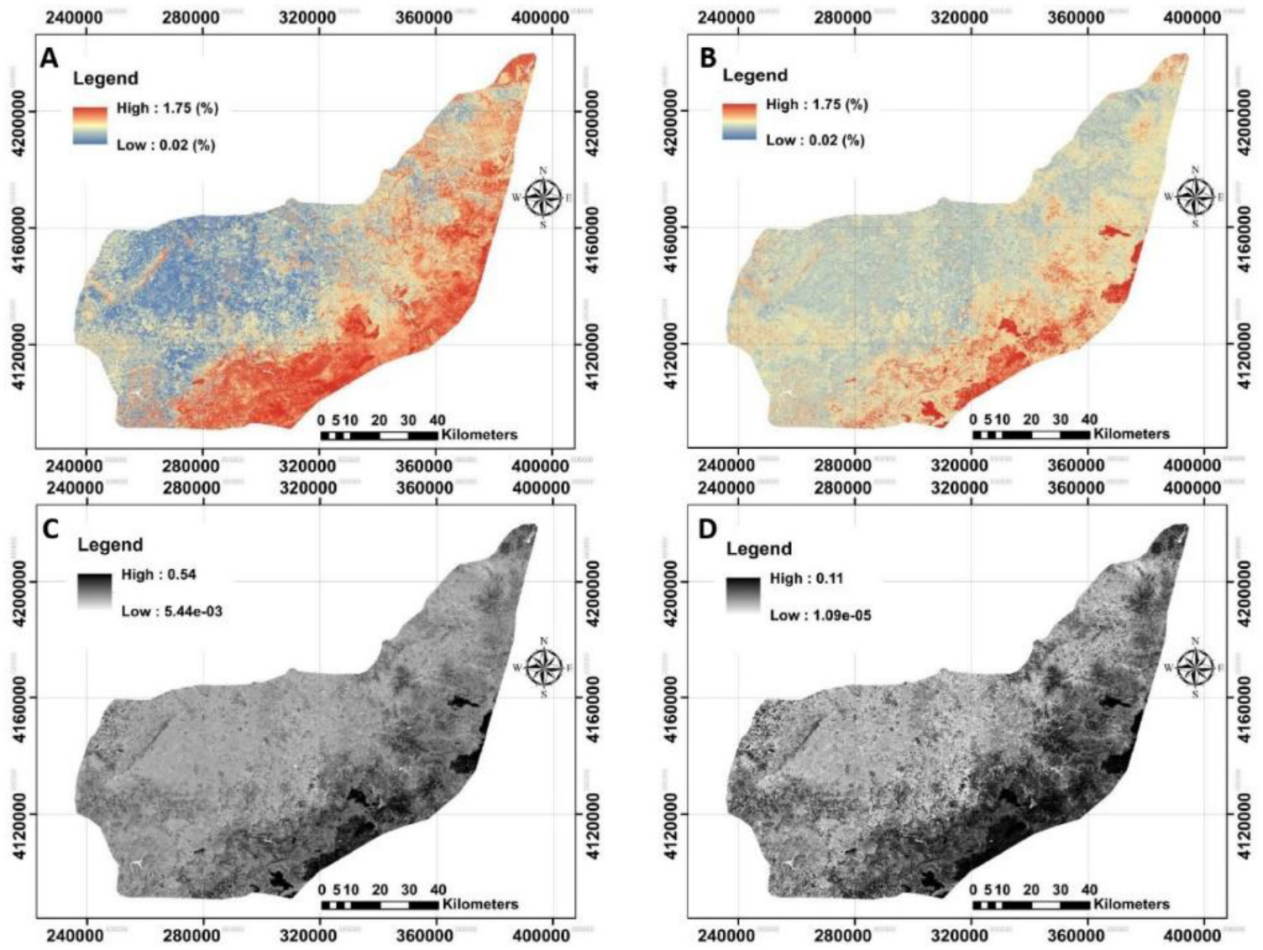
The provided digital maps of SOC25–50cm across the study area using RF model. **A**: Mean prediction (%); **B**: prediction of interval range (%); **C**: standard error map of the prediction; **D**: variance map of the prediction. (Own source).

**Figure 9.**
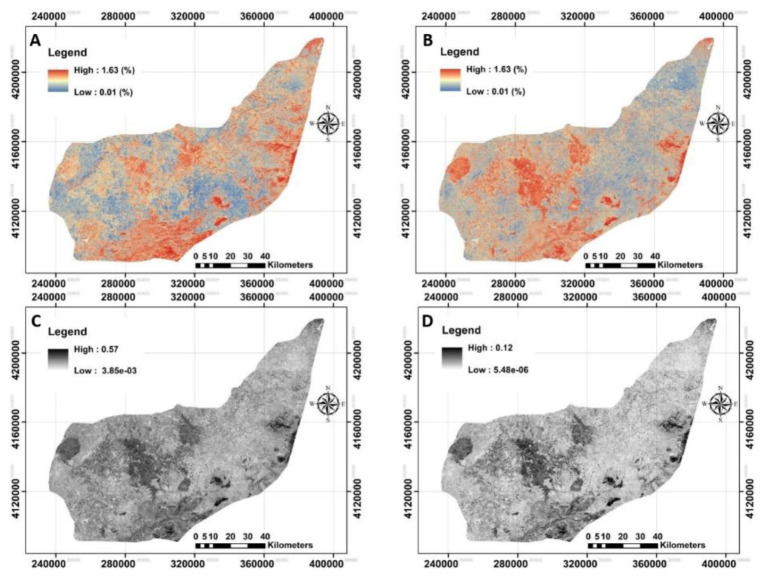
The provided digital maps of SOC50–75cm across the study area using RF model. **A**: Mean prediction (%); **B**: prediction of interval range (%); **C**: standard error map of the prediction; **D**: variance map of the prediction. (Own source).

### Digital maps and quantified uncertainties

The next step was to provide digital maps of SOC for three selected depths associated with their uncertainties using bootstrapping method ( Figure 7– Figure 9). The standard error and variance maps of predictions were also illustrated. 

Based on the provided digital maps ( Figure 6– Figure 8), the highest mean value of SOC was recorded for the SOC _0–25cm_ (0.79 g/100g, on average), followed by SOC _25–50cm_ (0.49 g/100g, on average) and SOC _50–75cm_ (0.32 g/100g, on average). A similar downward trend with depth had also been observed for the field data ( Table 2). These results generally show the good accuracy of the prepared maps.

The next step was to quantify the map’s accuracy using the bootstrapping method. The pixel-by-pixel values of standard error and variance at any depth were illustrated before calculating the prediction interval ranges ( Figure 7, Figure 8 and Figure 9). On contrary of our expectation, the highest PI was found in terms of SOC _0–25cm_ (0.44 g/100g, on average), while the lowest one was for SOC _50–75cm_ (0.24 g/100g, on average) indicating the low accuracy of top layers compared with the bottom ones. A possible reason may be that SOC is one of the most dynamic of soil properties ( Stolt *et al*., 2010). These results support that the SOC values in middle and deep soils are not affected by crop or farm activities ( Montes-Pulido *et al*., 2016; Zhu *et al*., 2017) and, for that, crop monitoring through satellites is feasible for monitoring SOC in topsoil. In general, the high values of uncertainties were found in the south of the study area.

## Conclusions

This study aims to reveal the usage of DSM and its importance in predicting and spatial distribution of SOC at three depths using machine learning techniques. In general, the results showed that random forest outperformed cubist in all predictions. Furthermore, the spatial distribution SOC was successfully mapped using integrated indices as a novel idea.

Although this research focuses on the mapping of SOC at three depths, there are at least two potential limitations concerning the results of this study. A first limitation concerns the absence of harmonised data for horizons based on the GlobalSoilMap projects. These lack of harmonised data is a barrier to develop comparative analysis between data sources so harmonisation and standardisation processes are required. A second potential limitation is that because there was no data on bulk density, the soil carbon storage was not calculated to monitor the climate change impact. We suggest using a time series of finer integrated remotely-sensed data in the future.

The analysis of SOC on different soil types allows enhancing of results. The terrain attributes analysis was a key step in order to obtain SOC data in middle depth. Taken together, our findings indicate the successful usage of machine learning with application of user-friendly software e.g., R programming.

The use of satellites for monitoring SOC trends is feasible for soil top layers due to crop impact on it. In addition, several agricultural policies and/or contract solutions such as new CAP (Common Agricultural Policies), Integrated production, Ecologic production and so on, may be beneficial for this methodology in order to reduce costs in hot-spot checks. Furthermore, the technology proposed may support the development of new contract solutions based on results within a payment per carbon sequestered.

## Ethics and consent

Ethical approval and consent were not required.

## Data Availability

The Landsat-8 OLI images used in the study are publicly available for download here: https://www.usgs.gov/landsat-missions/landsat-data-access. All pictures from July 2019 coincident to the study area were used. The Sentinel-2A MSI images used in the study are publicly available for download here: https://scihub.copernicus.eu/. All pictures from July 2019 coincident to the study area were used. The DEM, soil maps and climate variables used in this study are available on REDIAM: https://descargasrediam.cica.es/repo/s/RUR. The data can be found categorized by topic in 01_CARACTERIZACION_TERRITORIO/07_BASES_REF_ELEV (DEM data) and 16_INDICADORES_ESTADISTICAS/05_DATOS_BASICOS (climate data). The reader can download the data in several formats (Access, shapes, WMS, *etc*). SEISNET: https://microleis.evenor-tech.com/banco/seisnet/seisnet.htm In SEISNET platform, soil data can be downloaded at different details scale. T data used in this research work can be downloaded in the subsection ‘Nivel de información #3’ (registration is required). Open Science Framework: CONSOLE. https://doi.org/10.17605/OSF.IO/B7S4Z ( Velázquez, 2022). This project contains the following underlying data in the file *source_data.rar*: Rasters (indexes obtained from Sentinel and Landsat satellites). Field_data.xls (physical and chemical soil variables, climate variables and DEM obtained from REDIAM, SEISNET and ASAJA-SEVILLA used in this work). Data are available under the terms of the Creative Commons Zero "No rights reserved" data waiver (CC0 1.0 Public domain dedication).
