# Supplementary material for: The possibility of spatial mapping of SOC content in olive groves under integrated production using easy-to-obtain ancillary data in a Mediterranean area
Source: Open Res Eur. 2024 Jan 17;2:110. Originally published 2022 Sep 20. [Version 2] doi: 10.12688/openreseurope.14716.2 (PMC11069042; doi:10.12688/openreseurope.14716.2)
Supplement: Peer review history

# Reviewer report: Reviewer response for version 2

**DOI:** 10.21956/openreseurope.18038.r39750

**Published:** 2024-05-03

**Authors:** Odunayo David Adeniyi

**Affiliations:** 1 University of Pavia, Pavia, Italy

1. The R software version used should be cited. 

 2. In the Field sampling and soil analysis section, the total number of soil datasets used wasn't stated. 

 3. Still under the  Field sampling and soil analysis section, "The information compiled (physical and chemical soil variables (SEISNET and ASAJA-Sevilla); site, mean annual precipitation, temperature and evapotranspiration (REDIAM)) were harmonised using R Software (identifying potential duplicates and errors) so that they had the same resolution and extent for analysing the relationship between SOC and other variables in-situ and ex-situ. A total of 22 commercial olive groves included in the data compiled from ASAJA-Sevilla were selected, taking into account their representativeness in terms of soil types and climatic conditions." Kindly rewrite this paragraph so that it can be well understood. 

 4. In Statistical analysis section, kindly rewrite this paragraph. Maybe you shouldn't start with "Since...". Or maybe the section is not even needed. Kindly delete. 

 5. What is the name of the DEM (where did you get it from and Why such product?)

 6. The remote sensing data were gotten from July 2019, what is the percentage cloud cover condition on that day? Why this date? Does it coincide with the soil sampling date? Which day in July 2019?

 7. The Climate variables was gotten from where? How did you downscale this climatic variables from 100-m to 30-m resolution?

 8. ML techniques section: R programming (version 3.5.1), Good! kindly cite it.

 9. " Compared with the cubist, the difference between lower and upper quartiles in the RF is larger. Furthermore, RF tends to perform better in different iterations ( Zhou *et al*., 2019). Khaledian and Miller (2020) reported that the results taken by cubist are more interpretable than RFs’ which are semi-interpretable. We therefore performed both models, then one of them was selected as a parsimonious model to be applied in spatial mapping." I don't think this is needed here.

 10. How did you arrive at the hyperparameters you used for the RF and Cubist? did you perform hyperparameter tuning?

 11. In "Acquiring spatial maps" section: "In addition to the rich capacity of modelling in R, it has a user-friendly environment to map spatial data" There are other programming languages too that does same thing, such as Python, etc. I think this statement and others like this isn't needed. R is just a tool like Python and others...

 12. Interesting, to see that ggplot2 package was used for spatial mapping not 'raster' or 'terra', etc. Are you sure of this? Is the code attached to this article?

 13. The Results section. The Brief soil data didn't give us the explanatory/description of the soil data used for the modelling of the SOC. 

 14. This Terrain-related attributes section is not part of your result nor is it an objective of this article. It shouldn't be here. Maybe in the Materials and Method section. Same as the Remote-sensed data section.

 15. In the Final ML technique section: You need to discuss why RF outperformed Cubist model. 

 16. 'The results generally revealed that Landsat -based indices are not better predictors', How did you come to this conclusion? Maybe it would have been better if you have compare  Landsat -based indices + other ancillary variables versus Sentinel-based indices + other ancillary variables. 

 17. "In addition, the climate data were not as important as other supplied covariates", of course, they were originally in 100-m resolution. Such a coarse resolution might not fit to explain SOC variation at that high resolution. This doesn't mean climate couldn't be an important variable for SOC variability in this study area. Maybe you shouldn't have use a coarser climatic variable raster, or, instead, find a higher climatic raster. 

 19. You didn't describe the digital maps. Is it coherence with the Input variables? How can this maps be justify?

Is the study design appropriate and does the work have academic merit?

No

Is the work clearly and accurately presented and does it cite the current literature?

No

If applicable, is the statistical analysis and its interpretation appropriate?

No

Are all the source data underlying the results available to ensure full reproducibility?

Yes

Are the conclusions drawn adequately supported by the results?

No

Are sufficient details of methods and analysis provided to allow replication by others?

No

Reviewer Expertise:

Machine Learning, digital soil mapping, remote sensing

I confirm that I have read this submission and believe that I have an appropriate level of expertise to state that I do not consider it to be of an acceptable scientific standard, for reasons outlined above.

# Reviewer report: Reviewer response for version 2

**DOI:** 10.21956/openreseurope.18038.r39023

**Published:** 2024-04-29

**Authors:** Calogero Schillaci

**Affiliations:** 1 JRC European Commission, Ispra, Italy

Dear Authors,

 The manuscript "The possibility of spatial mapping of SOC content in olive groves under integrated production using easy-to-obtain ancillary data in a Mediterranean area" is relevant. However, some issues need to be addressed.

 I appreciate that you used Open Research EU as the outcome of your research. I suggest to modify the title slightly, the manuscript is a case study, title seems long and not so appealing. Sure, you can apply spatial mapping; the point is, what is the outcome of the case study, and why should Olive Grove have been different from another land cover (e.g. Cropland)? Which aspect can be highlighted from this experience (e.g., particular precaution in the sampling design, difficulties in sampling close to the plant stem, root system making the task challenging, or influence of the soil sample)? All these things deserve the same importance: the algorithm used and the data treatment. Algorithm hyperparameter tuning is fundamental, you can check several good papers report the algorithms and their hyperparameter search for several reasons, to prove that the algorithm learn well and that it does not overfit (see suggested reads, Schillaci C,et.al.,2021 [Ref 1] ,Veronesi F,et.al.,2019 [Ref 2] , Schillaci C,et.al.,2017 [Ref 3], Schillaci C,et.al., 2018 [Ref 4]). Please try to add all these missing parts. The decision to use the cubist and RF should be motivated (e.g. mostly used in SOC mapping, but to state this, you need to perform a literature analysis (see suggested read Schillaci C,et.al., 2018 [Ref 4] ) and collect evidence about the algorithm used in similar contexts, Mediterranean, olive grove or fruit trees...at least in croplands).

 The statement about the RS indices that are more relevant than other predictors is not so new (See suggested read Schillaci C,et.al.,2017 [Ref 3]).

 Please try to update this parts and provide the limitations of the proposed methodologies as well as future perspectives and lesson learned from this experiment.

 I provided the reference as suggested reads, no need to add this specific references, but look at them and references therein.

 Kind regards

Is the study design appropriate and does the work have academic merit?

Partly

Is the work clearly and accurately presented and does it cite the current literature?

Partly

If applicable, is the statistical analysis and its interpretation appropriate?

Yes

Are all the source data underlying the results available to ensure full reproducibility?

Yes

Are the conclusions drawn adequately supported by the results?

Partly

Are sufficient details of methods and analysis provided to allow replication by others?

Yes

Reviewer Expertise:

Agricultural and Environmental Sciences

I confirm that I have read this submission and believe that I have an appropriate level of expertise to confirm that it is of an acceptable scientific standard, however I have significant reservations, as outlined above.

## References

[1] : New pedotransfer approaches to predict soil bulk density using WoSIS soil data and environmental covariates in Mediterranean agro-ecosystems. *Sci Total Environ* .2021;780: 10.1016/j.scitotenv.2021.146609 146609 10.1016/j.scitotenv.2021.146609 34030315

[2] : Comparison between geostatistical and machine learning models as predictors of topsoil organic carbon with a focus on local uncertainty estimation. *Ecological Indicators* .2019;101: 10.1016/j.ecolind.2019.02.026 1032-1044 10.1016/j.ecolind.2019.02.026

[3] : Spatio-temporal topsoil organic carbon mapping of a semi-arid Mediterranean region: The role of land use, soil texture, topographic indices and the influence of remote sensing data to modelling. *Sci Total Environ* .2017;601-602: 10.1016/j.scitotenv.2017.05.239 821-832 10.1016/j.scitotenv.2017.05.239 28578240

[4] : Modelling of Soil Organic Carbon in the Mediterranean area: a systematic map. *Rendiconti Online della Società Geologica Italiana* .2018;46: 10.3301/ROL.2018.68 161-166 10.3301/ROL.2018.68

# Reviewer report: Reviewer response for version 1

**DOI:** 10.21956/openreseurope.15895.r32365

**Published:** 2024-05-03

**Authors:** Prava Kiran Dash

**Affiliations:** 1 Department of Soil Science, College of Agriculture, Odisha University of Agriculture and Technology, Bhubaneswar, Odisha, India

This article is a good attempt to make SOC maps for the said study area. Overall a good attempt, but needs improvements. Although not too novel in terms of methods, but still this kind of regional paper also needs to be published to encourage mapping attempts at a regional scale and also for the agricultural management of the concerned and similar areas. However, it needs some rectifications, deletions, and additions before indexing. I have observed a number of mistakes. I am mentioning my detailed observations below. The authors are requested to improve the manuscript before final indexing.

 Best wishes to the authors.

**Comments:**

**Title:**

- The title of the article can be modified: “the possibility” can be deleted.
- This study has used legacy soil data, so that should be mentioned in the title as well. (for example, spatial mapping of soil organic carbon content at three depths using legacy soil data and easy-to-obtain ancillary data in a Mediterranean area).
- Exact place name should be mentioned instead of Mediterranean area (for example, Andalucía in southern Spain).

**Abstract:**

- Unlike most of Europe, Andalucía in southern Spain as a Mediterranean area still lacks digital maps of soil organic carbon (SOC)--> Mediterranean area that still lacks
- Operational Land Imager ‘OLI’ and... -> Operational Land Imager (OLI).
- Multispectral Instrument ‘MSI’… -> Multispectral Instrument (MSI).
- goodness-of-fit criteria: please mention the exact parameters.
- “We obtained evidence that the resolution of satellite images is a key parameter in modelling and digital mapping” - was this the objective of the study to determine the importance of resolution? If yes, mention it in the title and elsewhere and frame the research question accordingly. Otherwise, I just suggest restructuring the conclusion part of the abstract.
- Based on the objectives mentioned in the last paragraph of introduction, the conclusion part should just answer those questions: 1) at what accuracy the maps were prepared. 2) what was the best algorithm/model 3) ranking of the predictors/covariates.

**Introduction:**

- The first paragraph of the introduction looks unnecessary.
- 2 ^nd^ paragraph: "DEM-derived data can save considerable labour and costs..." - explain how?
- "The spatial patterns of SOC were linked topography in a case ... "-> linked to topography.
- "Venter et al. (2021) relates elevation ..." -> related.
- "There are other research works with Landsat-8 OLI, Sentinel-2A and EnMap spectral bands to estimate soil organic matter (Rosero-Vlasova et al, 2019) in Brazil" - it says research works, which means multiple research should have been cited. Please reframe the sentence.
- "agriculturally important nutrients (Shahbazi et al., 2019a) and the soil ripening process in Iran (Mousavi et al., 2020); and available water content in Australia (Gooley et al., 2014)…" - keep only one ‘and’.
- Introduction and elsewhere: Abstract says both Seville and Cordoba provenience. But, in the introduction section, only Seville is mentioned. Please clarify/rectify.
- Last paragraph of introduction: "(Landsat-8 OLI, Sentinel-2A MSI) as well as integration and terrain-related attributes (DEM-derived data)…" -> (Landsat-8 OLI, Sentinel-2A MSI), as well as integration and terrain-related attributes (DEM-derived data)…
- "The information compiled (physical and chemical soil variables (SEISNET and ASAJA-Sevilla); site, mean annual precipitation, temperature and evapotranspiration (REDIAM)) were harmonized using R Software (identifying potential duplicates and errors) so that they had the same resolution and extent for analysing..." - Not clear, please reframe the sentences and use the punctuation marks properly for better understanding.

**Materials and methods:**

- Figure 2 is unnecessarily complicated. The second line of contents (C) are not at all required. From (A) and (B) remove the example of indices as they are mentioned in details in the tables. In D, shorten the sentences. No need to write full sentences. Remember this is just a flow diagram. The details mentioned in the paragraph format are enough for understanding. Simplify the arrows in Figure 2. It should look simple and less complicated.
- The softwares used for deriving the covariates can be added in an additional column of table 1.
- "Landsat-8 OLI and Sentinel-2A MSI images acquired in July 2019 (see Underlying data) were selected for further analysis in this project due to the high probability of no clouds in the images." Was this the reason for selecting these satellite images? Or, is there any scientific reason?
- "Fortunately, the selected scenes comprise minimal cloud coverage and maximum soil surface exposure." The correct way to write this is…XYZ satellite imageries were selected based on minimum cloud coverage percentage.
- "To explain the vegetation, soil and water, landscape as well as geology of the study area, six band ratios were calculated using both images as well as their integrations for each index. The hypothesis of integrating the indices has been raised from the report of Wang et al. (2020) which demonstrated that combining Landsat TM and ALOS PALSAR images could predict more accurate SOC content, especially for soils with high vegetation canopy density in Spain." What is the need of mentioning this sentence in between methodology. Please delete.
- "To predict the spatial distribution of SOC, it was found that despite topographic variables which had higher influence at finer scales, the climatic variables were more important at coarser scales e.g., sub-regional to global scale (Adhikari et al., 2020). Among the previously described indices, climate variables contributed the most to explaining SOC content variation (Zhou et al., 2020)." No need to repeat the sentences with the same meaning repeatedly. Please delete this and other unnecessary sentences.
- "machine learning (ML)": use the abbreviation only once at the beginning of the paper. No need to mention it again and again.
- “Regarding the accelerated adoption of machine learning (ML) techniques in prediction of soil properties, especially in the last 10 years (Padarian et al., 2020)”: Unnecessary, please delete.
- "...(composed by 20% of data no included in the modelling process)..." -> composed of; data **not **included.
- "e.g., concordance (CCC)...": Write the full form of CCC.
- "It is clear that R ^2^ measures the precision of the relationship (between observed and predicted) while concordance as a single statistic evaluates the accuracy and precision of the relationship." Delete ‘it is clear that’; single statistical parameter that evaluates; add a reference to the sentence.
- "Similar work was fully explained by Malone et al. (2017) using the “goof” function within the “ithir” package in R (Malone, 2016)." - similar work or the details regarding the parameters? Please clarify.
- “In addition to the rich capacity of modelling in R, it has a user-friendly environment to map spatial data (Malone et al., 2017). Bivand et al. (2013) documented some methods for handling spatial data and analysis in R in a detailed manner.” - Not required (may delete).
- "same projection in advance to be easily import..." -> to easily import.
- “Arrouays et al. (2020) reported that uncertainty maps may give very strong arguments in DSM because the identifying missing soil data and excessive field work will be distinguished.” - Not clear, please rectify or delete.

**Results:**

- alluvial episodes?
- Etc -> etc.
- "In fact, the impact of Integrated Production was analysed comparing SOC data previous Integrated Production implementation (2005) and current SOC data. The results showed the increase in SOC due to the implementation of Integrated Production in terms of crop and soil management. (Figure 3)." What is the purpose of giving this information and this figure? It is not the research question. Please stick to the objectives and don’t put information unnecessarily.
- The sub sections Terrain-related attributes and Remotely-sensed data in the results section is irrelevant. It could have been placed in the methods section, if it was supposed to be kept in the paper. Please move these contents to methods, else simply delete. This is not the results of this paper based on the objectives set (as mentioned in the introduction).
- "Final machine learning technique" - Please change this sub-section title.
- "Machine learning allowed us to analysis the huge dataset and their relevance to SOC at multiple depths. For that, the use of open software like as R facilitated modeling and then mapping." - Not needed, please delete (already discussed in methods).
- Where is the actual results paragraph? Please explain table 3 in details. This is the actual result of this study. At what accuracy the maps could be prepared? Discuss in detail CCC, RMSE for each depth, each model.
- "The results generally revealed..." -> Results revealed that...
- "Landsat -based indices" - delete the extra space.
- "The integrated indices e.g., NBR_ITG, NDWI_ITG and CMR_ITG were identified as the most important covariates in prediction of SOC0–25cm, SOC25–50cm and SOC50–75cm, respectively." - Figure 6 says something different. Please explain the 3 best predictors for each depth as presented in Fig. 6. Modify the paragraph. Read and match with the figure carefully.
- "In general, the high values of uncertainties were found in the south of the study area." - What could be a possible reason?

**Conclusion:**

- Limitations can be improved? Is only lack of data availability the only limitation? Try to minimize the limitations in the conclusion section. They can be added in the discussion part after improvisation.
- "The analysis of SOC on different soil types allows enhancing of results. The terrain attributes analysis was a key step in order to obtain SOC data in middle depth. Taken together, our findings indicate the successful usage of machine learning with application of user-friendly software e.g., R programming." - Not necessary, please delete.
- "The use of satellites for monitoring SOC..." -> The use of satellite imageries/satellite derived information.
- "In addition, several agricultural policies and/or contract solutions such as new CAP (Common Agricultural Policies), Integrated production, Ecologic production and so on, may be beneficial for this methodology in order to reduce costs in hot-spot checks. Furthermore, the technology proposed may support the development of new contract solutions based on results within a payment per carbon sequestered." - delete, not necessary. This is not the research question.
- In conclusion the authors should simply say XYZ was the research question and XYZ is the answer. In this paper, if the authors can simply say we found...XYZ model performed better, XYZ was the CCC, XYZ was the RMSE, this was the best predictor (for each depth), that would serve the purpose.

All the best.

Is the study design appropriate and does the work have academic merit?

Partly

Is the work clearly and accurately presented and does it cite the current literature?

Partly

If applicable, is the statistical analysis and its interpretation appropriate?

Yes

Are all the source data underlying the results available to ensure full reproducibility?

Yes

Are the conclusions drawn adequately supported by the results?

No

Are sufficient details of methods and analysis provided to allow replication by others?

Yes

Reviewer Expertise:

Digital soil mapping

I confirm that I have read this submission and believe that I have an appropriate level of expertise to confirm that it is of an acceptable scientific standard, however I have significant reservations, as outlined above.

# Reviewer report: Reviewer response for version 1

**DOI:** 10.21956/openreseurope.15895.r32370

**Published:** 2023-06-13

**Authors:** Fuat Kaya

**Affiliations:** 1 Isparta University of Applied Sciences, Isparta, Turkey

I have carefully examined the mentioned article that aims to efficiently model the soil organic carbon content across three different soil depths within a watershed using the DSM (Digital Soil Mapping) framework.

 The study does appear to be built on an unsound scientific foundation. Moreover, there are significant shortcomings in the title, abstract, and overall writing of the article.

 As can be understood, the manuscript compared the performance of machine learning (ML) algorithms with 2 different mathematical foundations (RF and Cubist). However, what were the specific reasons for choosing these two algorithms among the numerous alternative MLs available? While a comprehensive comparison of all algorithms may prove impractical, the rationale behind our particular choices must be explained.

 First of all, the aims of this study are not well defined to fill the gap in the research question and literature. There appears to be an enormous lack of hypotheses in the study. What did you assume? As you emphasized in the conclusion of the abstract, does the spatial resolution of satellite images make a significant difference in the estimation of soil SOC content at 3 depths? Such a hypothesis was studied in different regions and its results were presented. For example;

 --Do more detailed environmental covariates deliver more accurate soil maps? (Samuel-Rosa *et al.*, 2015 ^1^).

 --Effects of different sources and spatial resolutions of environmental covariates on predicting soil organic carbon using machine learning in a semi-arid region of Iran (Garosi *et al.*, 2022 ^2^).

 It will be very difficult to develop a research question or hypothesis that can advance scientific knowledge by bypassing this literature. What I understand from the article is an approach such as averaging two different satellite-based indices. An assumption about this would be innovative. However, it is necessary to emphasize both the spectral and spatial resolutions of the relevant sensors (MSI and OLI).

 Based on my thorough examination of the article, I will provide detailed explanations below. The authors will have put in considerable effort to salvage the article from being rejected. Consequently, I recommend reading this article to gain insights into potential areas of focus within DSM that could contribute to advancing scientific knowledge.

 --Ten challenges for the future of pedometrics (Wadoux *et al.*, 2021 ^3^).

 Furthermore, I suggest restructuring the article by formulating research questions that could lead to innovative solutions for addressing various challenges. As it stands, the current form of the article fails to offer anything beyond the application of existing DSM techniques commonly employed in the Mediterranean region.

**Title:** Consider replacing the title: "possibility" with something else.

**Abstract**: Your manuscripts focus on regression problems, which necessitates structuring the abstract to incorporate quantitative model evaluation measures such as model performance criteria (e.g., "RMSE," "MAE," or "R2") used in your study. It is important to acknowledge that these predictive maps inherently carry a degree of error.

 <<<< ‘MSI’ <<<<<<<<<<< (MSI)

 <<<< ‘OLI <<<<<<<<<<< (OLI)

 use parentheses to show abbreviations.

**Introduction**:

 The introduction is very technically written. DSM literature in these areas is overlooked, as is the SOC cycle in the Mediterranean and the challenges in SOC management.

**Material-Methods:**

 "Integrated production includes several rules (Romero-Gámez et al., 2017) for crop, soil, and water management to increase the sustainable production and other agri-environmental public goods, such as carbon sequestration, and promoting the reduction of fertilizers and pesticides".

 The sentences above do not seem appropriate to the section they are in.

**Field sampling and soil analysis:**

 What is the exact date of taken of the samples in 22 commercial olive orchards?

**Ancillary data:**

- It is important to include multiple digital environmental variables that may be associated with soil formation to explain the variation in the area. However, raw grid resolutions of each must be provided. Next, you need to specify that you are aggregating or disaggregating the spatial modeling framework. For example, when the raw resolutions of your variables vary between 10 and 100 meters, at what meter resolution would you put them in the same frame?
- Provide a proper reference to the R program (citation" ").
- Explanatory and transparent information about the hyperparameter optimization of either algorithm is not presented in manuscript as a supplement.
- I downloaded up to 3GB of your data. but I couldn't find any reproducible code. There are only "Rasters" and "Field_data". Turns out that you're not actually sharing reproducible results.

**Results and discussion:**

- When I come to the findings of the study, I am completely disappointed. Using very old data, how can we generate a digital map of SOC using RS data from 2019? I haven't seen this approach much in DSM. Of course, similar ones may have been published, but this is completely incomplete. Use the index values you get from the average band values of the satellite images from 1990-2000 (of course, no Sentinel 2 here) and the satellite images from 2000-2010 because you have a different data set. Otherwise, what you're doing is not guessing, it's giving the model a number and getting a number.
- Terrain-related attributes and Remotely-sensed data sections are placed to fill the page. There's no need for them.
- What is IncMSE in Figure 6? Don't leave everything to the reader. You need to provide information about this in the material method.
- What was the ability of the 22 commercial olive groves to represent the entire area? There is tremendous noise on forecast maps. How well do the environmental variables in the dataset match the whole study area?
- Is your work flawless, or are there any limitations in this study? Additionally, I would appreciate hearing your insights and recommendations for future research. It might be helpful to address these points as a separate paragraph within Section 3 of your work.

Is the study design appropriate and does the work have academic merit?

No

Is the work clearly and accurately presented and does it cite the current literature?

No

If applicable, is the statistical analysis and its interpretation appropriate?

No

Are all the source data underlying the results available to ensure full reproducibility?

No

Are the conclusions drawn adequately supported by the results?

No

Are sufficient details of methods and analysis provided to allow replication by others?

No

Reviewer Expertise:

Digitai soil mapping, machine learning, remote sensing

I confirm that I have read this submission and believe that I have an appropriate level of expertise to state that I do not consider it to be of an acceptable scientific standard, for reasons outlined above.

## References

[1] : Do more detailed environmental covariates deliver more accurate soil maps?. *Geoderma* .2015;243-244: 10.1016/j.geoderma.2014.12.017 214-227 10.1016/j.geoderma.2014.12.017

[2] : Effects of different sources and spatial resolutions of environmental covariates on predicting soil organic carbon using machine learning in a semi-arid region of Iran. *Geoderma Regional* .2022;29: 10.1016/j.geodrs.2022.e00513 10.1016/j.geodrs.2022.e00513

[3] : Ten challenges for the future of pedometrics. *Geoderma* .2021;401: 10.1016/j.geoderma.2021.115155 10.1016/j.geoderma.2021.115155

# Reviewer report: Reviewer response for version 1

**DOI:** 10.21956/openreseurope.15895.r30142

**Published:** 2022-10-05

**Authors:** Sameh Kotb Abd-Elmabod

**Affiliations:** 1 Soils & Water Use Department, Agricultural and Biological Research Division, National Research Centre, Cairo, Egypt

This work is good and interesting because it is based on using different datasets (soil database and Landsat-8 and Sentinel-2A images), and to run two models (random forest and Cubist) for the prediction of SOC at three soil depths. However, there are a few comments that are inserted below:

  

- The study area is located in Seville and Cordoba but you referred various times in the different manuscript sections only to prediction of SOC in southern Spain (Seville), so please go through the text and add the Sevilla and Cordoba provinces.

- In figure 1, the subfigure A should be improved and add the coordination, and subfigure 1D for which temperature, so please add mean temperature.

- In the flow chart you can use different colours for each step.

- In table 1, in the description of Normalized Difference Water Index, please put water instead of vegetation, and in the description of Clay Minerals Ratio and Normalized Difference Salinity Index, please put Soil instead of geology.

- In the section Terrain-related attributes, case study areas - change by study area.

- In my opinion you should separate the limitations of this study from the conclusion section. Maybe you can add a new section for the study limitations after the results and discussion. Also, one of the limitations is that the date of satellite images were recent (2019) but the dates of soil sampling were 1990–2019, why did you not use only the recent soil data (2019), as the soil organic carbon can be changed through time.

Is the study design appropriate and does the work have academic merit?

Yes

Is the work clearly and accurately presented and does it cite the current literature?

Yes

If applicable, is the statistical analysis and its interpretation appropriate?

Partly

Are all the source data underlying the results available to ensure full reproducibility?

Yes

Are the conclusions drawn adequately supported by the results?

Yes

Are sufficient details of methods and analysis provided to allow replication by others?

Yes

Reviewer Expertise:

Land evaluation, soil carbon, climate change

I confirm that I have read this submission and believe that I have an appropriate level of expertise to confirm that it is of an acceptable scientific standard.
